# Supplementary material for: Functional abilities of cultivable plant growth promoting bacteria associated with wheat (Triticum aestivum L.) crops
Source: Genet Mol Biol. 2016 Jan-Mar;39(1):111–21. doi: 10.1590/1678-4685-GMB-2015-0140 (PMC4807380; doi:10.1590/1678-4685-GMB-2015-0140)
Supplement: Supplementary file 1 [file 1415-4757-gmb-39-1-111-Suppl01.pdf]

Table S1 - Input dataset for MCA analysis.

[1]Name	[2]Sid	[3]Phos	[4]IC	[5]Genera
JC1	1	1	2	Variovorax sp.
JC2	1	1	1	Azospirillum sp.
JC3	2	1	2	Rhizobium sp.
JC4	2	1	1	Achromobacter sp.
JC5	2	1	3	Rhizobium sp.
JC6	2	1	2	Rhizobium sp.
JC7	1	1	1	Stenotrophomonas sp.
JC8	2	1	3	Rhizobium sp.
JC9	1	1	1	Ochrobactrum sp.
JC10	1	1	3	Pseudomonas sp.
JC11	2	1	2	Serratia sp.
JC12	1	1	1	Xanthomonas sp.
JC13	1	1	1	Luteibacter sp.
JC14	1	1	2	Rhizobium sp.
JC15	3	1	3	Pseudomonas sp.
JC16	2	1	3	Enterobacter sp.
JC17	2	1	3	Enterobacter sp.
JC18	2	1	2	Pseudomonas sp.
JC19	2	1	3	Enterobacter sp.
JC20	2	1	1	Rhizobium sp.
JC21	2	1	2	Cedecea sp.
JC22	2	1	3	Enterobacter sp.
JC23	2	1	3	Enterobacter sp.
JC24	2	1	1	Serratia sp.
JC25	2	1	2	Serratia sp.
JC26	2	1	2	Enterobacter sp.
JC27	2	1	1	Serratia sp.

JC28	2	1	3	Enterobacter sp.
JC29	2	1	3	Enterobacter sp.
JC30	2	1	3	Cedecea sp.
JC31	2	1	2	Pseudomonas sp.
JC32	2	1	1	Pseudomonas sp.
JC33	1	1	3	unidentified
JC34	2	1	1	unidentified
JC35	1	1	2	Rhizobium sp.
JC36	1	1	2	Rhizobium sp.
JC37	1	1	2	Rhizobium sp.
JC38	2	1	1	Pseudomonas sp.
JC39	1	1	2	Rhizobium sp.
JC40	2	1	2	Pseudomonas sp.
JC41	1	1	3	Azospirillum sp.
JC42	1	1	3	Azospirillum sp.
JC43	1	1	3	Azospirillum sp.
JC44	1	1	3	Azospirillum sp.
JC45	2	1	3	Enterobacter sp.
JC46	1	1	1	Azospirillum sp.
JC47	1	1	2	Azospirillum sp.
JC48	1	1	2	Pseudomonas sp.
JC49	1	1	2	Azospirillum sp.
JC50	1	1	1	Pedobacter sp.
JC51	1	1	2	Burkholderia sp.
JC52	1	2	1	Luteibacter sp.
JC53	2	1	1	Pandoraea sp.
JC55	2	1	2	Enterobacter sp.
JC56	2	1	3	Enterobacter sp.
JC57	2	2	3	Cedecea sp.
JC58	2	1	3	Cedecea sp.
JC59	1	2	1	Burkholderia sp.

JC60	2	1	2	Cedecea sp.
SB2	1	1	3	Rhizobium sp.
SB3	2	1	1	Pseudomonas sp.
SB4	1	1	2	Xanthomonas sp.
SB5	1	1	1	Rhizobium sp.
SB6	2	1	2	Pseudomonas sp.
SB7	2	1	3	Pseudomonas sp.
SB8	2	1	2	Pseudomonas sp.
SB9	2	1	2	Pseudomonas sp.
SB10	2	1	3	Pseudomonas sp.
SB11	2	1	1	Rhizobium sp.
SB12	2	1	2	Rhizobium sp.
SB13	1	1	1	Azospirillum sp.
SB14	1	1	2	Rhizobium sp.
SB15	2	1	1	Pseudomonas sp.
SB16	2	1	1	unidentified
SB17	2	1	1	Serratia sp.
SB18	2	1	2	Serratia sp.
SB19	2	1	1	Pseudomonas sp.
SB20	2	1	1	Pseudomonas sp.
SB21	2	1	1	Sphingomonas sp.
SB22	1	1	2	Leclercia sp.
SB23	1	1	2	Herbaspirillum sp.
SB24	1	1	2	Herbaspirillum sp.
SB25	2	1	3	Erwinia sp.
SB26	2	1	1	Serratia sp.
SB27	2	1	2	Serratia sp.
SB28	1	1	2	Herbaspirillum sp.
SB29	1	1	2	Herbaspirillum sp.
SB30	2	3	2	Serratia sp.
SB31	1	1	3	Pseudomonas sp.

SB32	2	1	1	Pseudomonas sp.
SB33	1	1	2	Rhizobium sp.
SB34	1	1	2	Rhizobium sp.
SB35	1	1	2	Leclercia sp.
SB36	1	1	2	Rhizobium sp.
SB37	2	1	1	Rhizobium sp.
SB38	1	1	2	Pseudomonas sp.
SB39	1	1	3	Rhizobium sp.
SB40	2	1	3	Pseudomonas sp.
SB41	2	1	1	Azospirillum sp.
SB42	1	1	1	Sphingomonas sp.
SB43	1	1	1	Azospirillum sp.
SB44	1	1	1	Azospirillum sp.
SB45	1	1	2	Herbaspirillum sp.
SB46	1	1	3	Caulobacter sp.
SB47	1	1	1	Azospirillum sp.
SB48	2	1	1	Pseudomonas sp.
SB49	2	1	3	Pseudomonas sp.
SB50	1	1	1	Xanthomonas sp.
SB51	2	1	1	unidentified
SB52	2	1	3	Cedecea sp.
SB53	2	1	3	Cedecea sp.
SB54	2	1	3	Leclercia sp.
SB55	2	1	3	Cedecea sp.
SB56	2	1	3	Leclercia sp.
SB57	2	1	3	Leclercia sp.
SB58	2	1	3	Leclercia sp.
SB59	2	1	3	Enterobacter sp.
SB60	2	1	3	Enterobacter sp.
VA1	2	1	2	Microbacterium sp.
VA2	1	1	2	Acinetobacter sp.

VA3	1	1	2	Sphingobium sp.
VA4	2	1	2	Azorhizobium sp.
VA5	2	1	1	Achromobacter sp.
VA6	1	1	1	Azospirillum sp.
VA7	1	1	1	Sphingomonas sp.
VA8	1	1	2	Leclercia sp.
VA9	2	1	1	unidentified
VA10	2	1	1	Pseudomonas sp.
VA11	2	3	3	Leclercia sp.
VA12	2	1	1	unidentified
VA13	2	1	1	Pseudomonas sp.
VA14	2	1	2	Burkholderia sp.
VA15	2	1	2	Azorhizobium sp.
VA16	1	1	2	Ochrobactrum sp.
VA17	1	1	1	Novosphingobium sp.
VA18	2	1	1	Enterobacter sp.
VA19	2	1	1	Pseudomonas sp.
VA20	2	2	1	Serratia sp.
VA21	2	2	1	Hafnia sp.
VA23	2	2	1	unidentified
VA24	2	2	1	unidentified
VA25	2	3	2	Hafnia sp.
VA26	2	2	1	unidentified
VA27	2	2	1	Hafnia sp.
VA28	2	2	2	Raoultella sp.
VA29	2	2	1	Hafnia sp.
VA30	2	2	1	Hafnia sp.
VA31	2	1	1	Azorhizobium sp.
VA32	2	1	2	Rhizobium sp.
VA33	2	1	1	Stenotrophomonas sp.
VA35	2	1	2	Rhizobium sp.

VA36	1	1	2	Rhizobium sp.
VA37	2	2	3	Pantoea sp.
VA38	1	1	1	Lysobacter sp.
VA39	2	1	1	Azorhizobium sp.
VA40	2	1	1	Pseudomonas sp.
VA41	2	1	2	Ochrobactrum sp.
VA42	1	2	2	Rhizobium sp.
VA43	2	1	2	unidentified
VA44	2	1	2	Rhizobium sp.
VA45	2	1	1	unidentified
VA46	2	1	1	unidentified
VA47	2	2	2	unidentified
VA48	2	2	1	Burkholderia sp.
VA49	2	1	2	unidentified
VA50	2	2	3	Pantoea sp.
VA51	2	3	2	Acinetobacter sp.
VA52	2	1	1	Pseudomonas sp.
VA53	2	1	1	Burkholderia sp.
VA54	2	1	2	Serratia sp.
VA55	2	1	1	Serratia sp.
VA56	2	3	1	Burkholderia sp.
VA57	2	1	1	Serratia sp.
VA58	2	1	2	Rhizobium sp.
VA59	2	3	1	Burkholderia sp.
VA60	1	1	2	Dyella sp.
CM1	3	1	2	Stenotrophomonas sp.
CM2	2	1	2	Pseudomonas sp.
CM3	2	1	2	Pseudomonas sp.
CM4	2	1	2	Pseudomonas sp.
CM5	3	1	2	Stenotrophomonas sp.
CM6	2	1	2	Pseudomonas sp.

CM7	3	1	2	Pseudomonas sp.
CM8	3	1	2	Pseudomonas sp.
CM9	3	1	2	Pseudomonas sp.
CM10	3	1	2	Pseudomonas sp.
CM11	1	1	2	Pseudomonas sp.
CM12	1	1	2	Pseudomonas sp.
CM13	1	1	2	Pseudomonas sp.
CM14	1	1	1	Herbaspirillum sp.
CM15	2	1	2	Pseudomonas sp.
CM16	3	1	2	Pseudomonas sp.
CM17	3	1	2	Pseudomonas sp.
CM18	2	1	2	Pseudomonas sp.
CM19	2	1	2	Pseudomonas sp.
CM20	2	1	3	Stenotrophomonas sp.
CM21	3	2	2	Burkholderia sp.
CM22	3	2	2	Burkholderia sp.
CM23	3	2	2	Burkholderia sp.
CM24	3	2	2	Burkholderia sp.
CM25	2	1	1	Burkholderia sp.
CM26	3	2	2	Burkholderia sp.
CM27	3	2	2	Burkholderia sp.
CM28	1	1	3	Enterobacter sp.
CM29	2	1	2	Burkholderia sp.
CM30	3	2	2	Burkholderia sp.
CM6.1	3	1	3	Pseudomonas sp.
CM19.1	2	1	2	Pseudomonas sp.
CM31	1	1	2	Stenotrophomonas sp.
CM32	1	1	2	Achromobacter sp.
CM33	1	1	2	Microbacterium sp.
CM34	1	1	2	Microbacterium sp.
CM35	1	1	2	Microbacterium sp.

CM36	1	1	2	Pseudomonas sp.
CM37	1	1	2	unidentified
CM38	2	1	2	Pseudomonas sp.
CM39	1	1	3	Pseudomonas sp.
CM40	2	1	3	Pseudomonas sp.
CM41	1	1	2	Burkholderia sp.
CM42	1	1	1	Burkholderia sp.
CM43	3	2	2	Burkholderia sp.
CM44	3	2	2	Burkholderia sp.
CM45	1	1	1	Burkholderia sp.
CM46	3	2	2	Burkholderia sp.
CM47	1	1	2	Burkholderia sp.
CM48	1	1	1	Burkholderia sp.
CM49	1	1	2	Dyella sp.
CM50	1	1	2	Burkholderia sp.
CM51	3	2	2	Burkholderia sp.
CM52	3	2	3	Burkholderia sp.
CM54	3	2	2	Burkholderia sp.
CM55	1	1	1	Burkholderia sp.
CM57	1	1	1	Burkholderia sp.
CM58	3	2	1	Burkholderia sp.
CM59	1	1	1	Burkholderia sp.
CM33.1	1	1	3	Rhizobium sp.
CM52.1	3	2	2	Burkholderia sp.
GM1	1	1	2	Stenotrophomonas sp.
GM2	1	1	2	Stenotrophomonas sp.
GM3	2	1	2	Stenotrophomonas sp.
GM4	2	1	3	Pseudomonas sp.
GM5	1	1	2	Pseudomonas sp.
GM6	1	1	2	Stenotrophomonas sp.
GM7	1	1	2	Achromobacter sp.

GM8	2	1	3	Pseudomonas sp.
GM9	1	1	2	Pseudomonas sp.
GM10	1	1	2	Stenotrophomonas sp.
GM11	3	2	2	Klebsiella sp.
GM12	3	2	2	Klebsiella sp.
GM13	1	1	3	Pseudomonas sp.
GM14	2	1	3	Enterobacter sp.
GM15	1	1	2	Rhizobium sp.
GM16	1	1	2	Pseudomonas sp.
GM17	3	1	2	Enterobacter sp.
GM18	3	1	3	unidentified
GM19	2	1	3	Pseudomonas sp.
GM20	1	1	3	Pseudomonas sp.
GM22	2	1	2	Burkholderia sp.
GM23	2	1	2	Klebsiella sp.
GM24	1	2	2	Burkholderia sp.
GM25	1	2	3	Burkholderia sp.
GM26	3	1	3	Burkholderia sp.
GM27	2	1	2	Burkholderia sp.
GM29	2	1	2	Burkholderia sp.
GM30	1	1	2	Burkholderia sp.
GM31	2	1	2	Pseudomonas sp.
GM32	2	1	2	Pseudomonas sp.
GM33	2	1	2	Enterobacter sp.
GM34	2	1	2	Citrobacter sp.
GM35	2	1	3	Pseudomonas sp.
GM36	2	1	2	Pseudomonas sp.
GM37	1	1	3	Pseudomonas sp.
GM38	1	1	2	Pseudomonas sp.
GM39	1	1	2	Pseudomonas sp.
GM40	1	1	2	Pseudomonas sp.

GM41	2	1	3	Pseudomonas sp.
GM42	1	1	3	Chryseobacterium sp.
GM43	1	1	2	Pseudomonas sp.
GM44	1	1	3	Pseudomonas sp.
GM45	2	2	2	Burkholderia sp.
GM46	1	1	2	Stenotrophomonas sp.
GM47	1	1	2	Pseudomonas sp.
GM48	1	1	2	Pseudomonas sp.
GM49	1	1	2	Pseudomonas sp.
GM50	1	1	2	Luteibacter sp.
GM51	3	2	1	Klebsiella sp.
GM53	1	2	2	Burkholderia sp.
GM54	1	2	2	Burkholderia sp.
GM55	1	1	2	Burkholderia sp.
GM57	1	1	2	Burkholderia sp.
GM58	1	1	2	Rhizobium sp.
GM59	3	1	2	Pantoea sp.
GM60	1	1	1	Luteibacter sp.
GM40.1	1	1	2	unidentified
GM45.1	3	2	2	unidentified
BV1	2	1	1	Stenotrophomonas sp.
BV2	1	1	1	Achromobacter sp.
BV3	1	1	2	Pseudomonas sp.
BV4	2	1	1	Stenotrophomonas sp.
BV5	1	1	2	Stenotrophomonas sp.
BV6	1	1	1	Pseudomonas sp.
BV7	2	1	1	Pseudomonas sp.
BV8	1	1	2	Xanthomonas sp.
BV9	1	1	2	Pseudomonas sp.
BV10	3	1	3	Salmonella sp.
BV11	1	1	2	Herbaspirillum sp.

BV12	1	1	3	Pseudomonas sp.
BV13	1	1	3	Herbaspirillum sp.
BV14	1	1	3	Herbaspirillum sp.
BV15	3	1	2	Xanthomonas sp.
BV16	1	1	1	Enterobacter sp.
BV17	1	1	2	Enterobacter sp.
BV18	3	1	1	Xanthomonas sp.
BV20	1	1	2	Herbaspirillum sp.
BV21	3	1	3	Pantoea sp.
BV22	3	3	2	Enterobacter sp.
BV23	2	1	3	Shigella sp.
BV24	2	1	2	Shigella sp.
BV25	2	1	2	Shigella sp.
BV26	2	1	2	Pantoea sp.
BV27	2	1	2	Pantoea sp.
BV28	2	1	1	Pantoea sp.
BV29	1	1	2	Shigella sp.
BV30	2	1	1	Shigella sp.
BV23.1	3	3	3	Enterobacter sp.
BV31	1	1	2	Chryseobacterium sp.
BV32	3	1	2	Pseudomonas sp.
BV33	2	1	2	Pantoea sp.
BV34	3	1	3	Salmonella sp.
BV35	1	1	3	Enterobacter sp.
BV36	3	1	3	Kluyvera sp.
BV37	1	3	3	Salmonella sp.
BV38	1	3	2	Salmonella sp.
BV40	3	1	3	Enterobacter sp.
BV43	2	1	2	Pantoea sp.
BV45	1	1	1	Burkholderia sp.
BV47	1	1	1	Burkholderia sp.

BV49	3	1	2	Burkholderia sp.
BV50	2	1	2	Dyella sp.
BV52	2	1	2	Burkholderia sp.
BV53	2	3	2	Burkholderia sp.
BV56	3	1	2	Burkholderia sp.
BV57	3	3	1	Burkholderia sp.
BV59	3	3	2	Enterobacter sp.
BV60	3	3	3	Burkholderia sp.
BV40.1	3	1	3	Enterobacter sp.

[1]Name: isolate identification.

[2]IC: indolic compounds production level.

[3]Phos: phosphate solubilization level.

[4]Sid: siderophores production level.

[5]Genera: according to partial 16S rRNA gene sequencing.
