# Supplementary material for: Functional abilities of cultivable plant growth promoting bacteria associated with wheat (Triticum aestivum L.) crops
Source: Genet Mol Biol. 2016 Jan-Mar;39(1):111–21. doi: 10.1590/1678-4685-GMB-2015-0140 (PMC4807380; doi:10.1590/1678-4685-GMB-2015-0140)
Supplement: Supplementary file 2 [file 1415-4757-gmb-39-1-111-Suppl02.pdf]

Table S2 - Input dataset for CatPCA analysis.

| [1]Name | [2]Niche | [3]Sampling Site | [4]Genera |
|---|---|---|---|
| JC1 | 1 | JC | Variovorax sp. |
| JC2 | 1 | JC | Azospirillum sp. |
| JC3 | 1 | JC | Rhizobium sp. |
| JC4 | 1 | JC | Achromobacter sp. |
| JC5 | 1 | JC | Rhizobium sp. |
| JC6 | 1 | JC | Rhizobium sp. |
| JC7 | 1 | JC | Stenotrophomonas sp. |
| JC8 | 1 | JC | Rhizobium sp. |
| JC9 | 1 | JC | Ochrobactrum sp. |
| JC10 | 1 | JC | Pseudomonas sp. |
| JC11 | 1 | JC | Serratia sp. |
| JC12 | 1 | JC | Xanthomonas sp. |
| JC13 | 1 | JC | Luteibacter sp. |
| JC14 | 1 | JC | Rhizobium sp. |
| JC15 | 1 | JC | Pseudomonas sp. |
| JC16 | 1 | JC | Enterobacter sp. |
| JC17 | 1 | JC | Enterobacter sp. |
| JC18 | 1 | JC | Pseudomonas sp. |
| JC19 | 1 | JC | Enterobacter sp. |
| JC20 | 1 | JC | Rhizobium sp. |
| JC21 | 1 | JC | Cedecea sp. |
| JC22 | 1 | JC | Enterobacter sp. |
| JC23 | 1 | JC | Enterobacter sp. |
| JC24 | 1 | JC | Serratia sp. |
| JC25 | 1 | JC | Serratia sp. |
| JC26 | 1 | JC | Enterobacter sp. |
| JC27 | 1 | JC | Serratia sp. |

| | | | |
|---|---|---|---|
| JC28 | 1 | JC | Enterobacter sp. |
| JC29 | 1 | JC | Enterobacter sp. |
| JC30 | 1 | JC | Cedecea sp. |
| JC31 | 2 | JC | Pseudomonas sp. |
| JC32 | 2 | JC | Pseudomonas sp. |
| JC33 | 2 | JC | unidentified |
| JC34 | 2 | JC | unidentified |
| JC35 | 2 | JC | Rhizobium sp. |
| JC36 | 2 | JC | Rhizobium sp. |
| JC37 | 2 | JC | Rhizobium sp. |
| JC38 | 2 | JC | Pseudomonas sp. |
| JC39 | 2 | JC | Rhizobium sp. |
| JC40 | 2 | JC | Pseudomonas sp. |
| JC41 | 2 | JC | Azospirillum sp. |
| JC42 | 2 | JC | Azospirillum sp. |
| JC43 | 2 | JC | Azospirillum sp. |
| JC44 | 2 | JC | Azospirillum sp. |
| JC45 | 2 | JC | Enterobacter sp. |
| JC46 | 2 | JC | Azospirillum sp. |
| JC47 | 2 | JC | Azospirillum sp. |
| JC48 | 2 | JC | Pseudomonas sp. |
| JC49 | 2 | JC | Azospirillum sp. |
| JC50 | 2 | JC | Pedobacter sp. |
| JC51 | 2 | JC | Burkholderia sp. |
| JC52 | 2 | JC | Luteibacter sp. |
| JC53 | 2 | JC | Pandoraea sp. |
| JC55 | 2 | JC | Enterobacter sp. |
| JC56 | 2 | JC | Enterobacter sp. |
| JC57 | 2 | JC | Cedecea sp. |
| JC58 | 2 | JC | Cedecea sp. |
| JC59 | 2 | JC | Burkholderia sp. |
| JC60 | 2 | JC | Cedecea sp. |

| | | | |
|---|---|---|---|
| SB2 | 1 | SB | Rhizobium sp. |
| SB3 | 1 | SB | Pseudomonas sp. |
| SB4 | 1 | SB | Xanthomonas sp. |
| SB5 | 1 | SB | Rhizobium sp. |
| SB6 | 1 | SB | Pseudomonas sp. |
| SB7 | 1 | SB | Pseudomonas sp. |
| SB8 | 1 | SB | Pseudomonas sp. |
| SB9 | 1 | SB | Pseudomonas sp. |
| SB10 | 1 | SB | Pseudomonas sp. |
| SB11 | 1 | SB | Rhizobium sp. |
| SB12 | 1 | SB | Rhizobium sp. |
| SB13 | 1 | SB | Azospirillum sp. |
| SB14 | 1 | SB | Rhizobium sp. |
| SB15 | 1 | SB | Pseudomonas sp. |
| SB16 | 1 | SB | unidentified |
| SB17 | 1 | SB | Serratia sp. |
| SB18 | 1 | SB | Serratia sp. |
| SB19 | 1 | SB | Pseudomonas sp. |
| SB20 | 1 | SB | Pseudomonas sp. |
| SB21 | 1 | SB | Sphingomonas sp. |
| SB22 | 1 | SB | Leclercia sp. |
| SB23 | 1 | SB | Herbaspirillum sp. |
| SB24 | 1 | SB | Herbaspirillum sp. |
| SB25 | 1 | SB | Erwinia sp. |
| SB26 | 1 | SB | Serratia sp. |
| SB27 | 1 | SB | Serratia sp. |
| SB28 | 1 | SB | Herbaspirillum sp. |
| SB29 | 1 | SB | Herbaspirillum sp. |
| SB30 | 1 | SB | Serratia sp. |
| SB31 | 2 | SB | Pseudomonas sp. |
| SB32 | 2 | SB | Pseudomonas sp. |
| SB33 | 2 | SB | Rhizobium sp. |

| | | | |
|---|---|---|---|
| SB34 | 2 | SB | Rhizobium sp. |
| SB35 | 2 | SB | Leclercia sp. |
| SB36 | 2 | SB | Rhizobium sp. |
| SB37 | 2 | SB | Rhizobium sp. |
| SB38 | 2 | SB | Pseudomonas sp. |
| SB39 | 2 | SB | Rhizobium sp. |
| SB40 | 2 | SB | Pseudomonas sp. |
| SB41 | 2 | SB | Azospirillum sp. |
| SB42 | 2 | SB | Sphingomonas sp. |
| SB43 | 2 | SB | Azospirillum sp. |
| SB44 | 2 | SB | Azospirillum sp. |
| SB45 | 2 | SB | Herbaspirillum sp. |
| SB46 | 2 | SB | Caulobacter sp. |
| SB47 | 2 | SB | Azospirillum sp. |
| SB48 | 2 | SB | Pseudomonas sp. |
| SB49 | 2 | SB | Pseudomonas sp. |
| SB50 | 2 | SB | Xanthomonas sp. |
| SB51 | 2 | SB | unidentified |
| SB52 | 2 | SB | Cedecea sp. |
| SB53 | 2 | SB | Cedecea sp. |
| SB54 | 2 | SB | Leclercia sp. |
| SB55 | 2 | SB | Cedecea sp. |
| SB56 | 2 | SB | Leclercia sp. |
| SB57 | 2 | SB | Leclercia sp. |
| SB58 | 2 | SB | Leclercia sp. |
| SB59 | 2 | SB | Enterobacter sp. |
| SB60 | 2 | SB | Enterobacter sp. |
| VA1 | 1 | VA | Microbacterium sp. |
| VA2 | 1 | VA | Acinetobacter sp. |
| VA3 | 1 | VA | Sphingobium sp. |
| VA4 | 1 | VA | Azorhizobium sp. |
| VA5 | 1 | VA | Achromobacter sp. |

| | | | |
|---|---|---|---|
| VA6 | 1 | VA | Azospirillum sp. |
| VA7 | 1 | VA | Sphingomonas sp. |
| VA8 | 1 | VA | Leclercia sp. |
| VA9 | 1 | VA | unidentified |
| VA10 | 1 | VA | Pseudomonas sp. |
| VA11 | 1 | VA | Leclercia sp. |
| VA12 | 1 | VA | unidentified |
| VA13 | 1 | VA | Pseudomonas sp. |
| VA14 | 1 | VA | Burkholderia sp. |
| VA15 | 1 | VA | Azorhizobium sp. |
| VA16 | 1 | VA | Ochrobactrum sp. |
| VA17 | 1 | VA | Novosphingobium sp. |
| VA18 | 1 | VA | Enterobacter sp. |
| VA19 | 1 | VA | Pseudomonas sp. |
| VA20 | 1 | VA | Serratia sp. |
| VA21 | 1 | VA | Hafnia sp. |
| VA23 | 1 | VA | unidentified |
| VA24 | 1 | VA | unidentified |
| VA25 | 1 | VA | Hafnia sp. |
| VA26 | 1 | VA | unidentified |
| VA27 | 1 | VA | Hafnia sp. |
| VA28 | 1 | VA | Raoultella sp. |
| VA29 | 1 | VA | Hafnia sp. |
| VA30 | 1 | VA | Hafnia sp. |
| VA31 | 2 | VA | Azorhizobium sp. |
| VA32 | 2 | VA | Rhizobium sp. |
| VA33 | 2 | VA | Stenotrophomonas sp. |
| VA35 | 2 | VA | Rhizobium sp. |
| VA36 | 2 | VA | Rhizobium sp. |
| VA37 | 2 | VA | Pantoea sp. |
| VA38 | 2 | VA | Lysobacter sp. |
| VA39 | 2 | VA | Azorhizobium sp. |

| | | | |
|---|---|---|---|
| VA40 | 2 | VA | Pseudomonas sp. |
| VA41 | 2 | VA | Ochrobactrum sp. |
| VA42 | 2 | VA | Rhizobium sp. |
| VA43 | 2 | VA | unidentified |
| VA44 | 2 | VA | Rhizobium sp. |
| VA45 | 2 | VA | unidentified |
| VA46 | 2 | VA | unidentified |
| VA47 | 2 | VA | unidentified |
| VA48 | 2 | VA | Burkholderia sp. |
| VA49 | 2 | VA | unidentified |
| VA50 | 2 | VA | Pantoea sp. |
| VA51 | 2 | VA | Acinetobacter sp. |
| VA52 | 2 | VA | Pseudomonas sp. |
| VA53 | 2 | VA | Burkholderia sp. |
| VA54 | 2 | VA | Serratia sp. |
| VA55 | 2 | VA | Serratia sp. |
| VA56 | 2 | VA | Burkholderia sp. |
| VA57 | 2 | VA | Serratia sp. |
| VA58 | 2 | VA | Rhizobium sp. |
| VA59 | 2 | VA | Burkholderia sp. |
| VA60 | 2 | VA | Dyella sp. |
| CM1 | 1 | CM | Stenotrophomonas sp. |
| CM2 | 1 | CM | Pseudomonas sp. |
| CM3 | 1 | CM | Pseudomonas sp. |
| CM4 | 1 | CM | Pseudomonas sp. |
| CM5 | 1 | CM | Stenotrophomonas sp. |
| CM6 | 1 | CM | Pseudomonas sp. |
| CM7 | 1 | CM | Pseudomonas sp. |
| CM8 | 1 | CM | Pseudomonas sp. |
| CM9 | 1 | CM | Pseudomonas sp. |
| CM10 | 1 | CM | Pseudomonas sp. |
| CM11 | 1 | CM | Pseudomonas sp. |

| | | | |
|---|---|---|---|
| CM12 | 1 | CM | Pseudomonas sp. |
| CM13 | 1 | CM | Pseudomonas sp. |
| CM14 | 1 | CM | Herbaspirillum sp. |
| CM15 | 1 | CM | Pseudomonas sp. |
| CM16 | 1 | CM | Pseudomonas sp. |
| CM17 | 1 | CM | Pseudomonas sp. |
| CM18 | 1 | CM | Pseudomonas sp. |
| CM19 | 1 | CM | Pseudomonas sp. |
| CM20 | 1 | CM | Stenotrophomonas sp. |
| CM21 | 1 | CM | Burkholderia sp. |
| CM22 | 1 | CM | Burkholderia sp. |
| CM23 | 1 | CM | Burkholderia sp. |
| CM24 | 1 | CM | Burkholderia sp. |
| CM25 | 1 | CM | Burkholderia sp. |
| CM26 | 1 | CM | Burkholderia sp. |
| CM27 | 1 | CM | Burkholderia sp. |
| CM28 | 1 | CM | Enterobacter sp. |
| CM29 | 1 | CM | Burkholderia sp. |
| CM30 | 1 | CM | Burkholderia sp. |
| CM6,1 | 1 | CM | Pseudomonas sp. |
| CM19.1 | 1 | CM | Pseudomonas sp. |
| CM31 | 2 | CM | Stenotrophomonas sp. |
| CM32 | 2 | CM | Achromobacter sp. |
| CM33 | 2 | CM | Microbacterium sp. |
| CM34 | 2 | CM | Microbacterium sp. |
| CM35 | 2 | CM | Microbacterium sp. |
| CM36 | 2 | CM | Pseudomonas sp. |
| CM37 | 2 | CM | unidentified |
| CM38 | 2 | CM | Pseudomonas sp. |
| CM39 | 2 | CM | Pseudomonas sp. |
| CM40 | 2 | CM | Pseudomonas sp. |
| CM41 | 2 | CM | Burkholderia sp. |

| | | | |
|---|---|---|---|
| CM42 | 2 | CM | Burkholderia sp. |
| CM43 | 2 | CM | Burkholderia sp. |
| CM44 | 2 | CM | Burkholderia sp. |
| CM45 | 2 | CM | Burkholderia sp. |
| CM46 | 2 | CM | Burkholderia sp. |
| CM47 | 2 | CM | Burkholderia sp. |
| CM48 | 2 | CM | Burkholderia sp. |
| CM49 | 2 | CM | Dyella sp. |
| CM50 | 2 | CM | Burkholderia sp. |
| CM51 | 2 | CM | Burkholderia sp. |
| CM52 | 2 | CM | Burkholderia sp. |
| CM54 | 2 | CM | Burkholderia sp. |
| CM55 | 2 | CM | Burkholderia sp. |
| CM57 | 2 | CM | Burkholderia sp. |
| CM58 | 2 | CM | Burkholderia sp. |
| CM59 | 2 | CM | Burkholderia sp. |
| CM33.1 | 2 | CM | Rhizobium sp. |
| CM52.1 | 2 | CM | Burkholderia sp. |
| GM1 | 1 | GM | Stenotrophomonas sp. |
| GM2 | 1 | GM | Stenotrophomonas sp. |
| GM3 | 1 | GM | Stenotrophomonas sp. |
| GM4 | 1 | GM | Pseudomonas sp. |
| GM5 | 1 | GM | Pseudomonas sp. |
| GM6 | 1 | GM | Stenotrophomonas sp. |
| GM7 | 1 | GM | Achromobacter sp. |
| GM8 | 1 | GM | Pseudomonas sp. |
| GM9 | 1 | GM | Pseudomonas sp. |
| GM10 | 1 | GM | Stenotrophomonas sp. |
| GM11 | 1 | GM | Klebsiella sp. |
| GM12 | 1 | GM | Klebsiella sp. |
| GM13 | 1 | GM | Pseudomonas sp. |
| GM14 | 1 | GM | Enterobacter sp. |

| | | | |
|---|---|---|---|
| GM15 | 1 | GM | Rhizobium sp. |
| GM16 | 1 | GM | Pseudomonas sp. |
| GM17 | 1 | GM | Enterobacter sp. |
| GM18 | 1 | GM | unidentified |
| GM19 | 1 | GM | Pseudomonas sp. |
| GM20 | 1 | GM | Pseudomonas sp. |
| GM22 | 1 | GM | Burkholderia sp. |
| GM23 | 1 | GM | Klebsiella sp. |
| GM24 | 1 | GM | Burkholderia sp. |
| GM25 | 1 | GM | Burkholderia sp. |
| GM26 | 1 | GM | Burkholderia sp. |
| GM27 | 1 | GM | Burkholderia sp. |
| GM29 | 1 | GM | Burkholderia sp. |
| GM30 | 1 | GM | Burkholderia sp. |
| GM31 | 2 | GM | Pseudomonas sp. |
| GM32 | 2 | GM | Pseudomonas sp. |
| GM33 | 2 | GM | Enterobacter sp. |
| GM34 | 2 | GM | Citrobacter sp. |
| GM35 | 2 | GM | Pseudomonas sp. |
| GM36 | 2 | GM | Pseudomonas sp. |
| GM37 | 2 | GM | Pseudomonas sp. |
| GM38 | 2 | GM | Pseudomonas sp. |
| GM39 | 2 | GM | Pseudomonas sp. |
| GM40 | 2 | GM | Pseudomonas sp. |
| GM41 | 2 | GM | Pseudomonas sp. |
| GM42 | 2 | GM | Chryseobacterium sp. |
| GM43 | 2 | GM | Pseudomonas sp. |
| GM44 | 2 | GM | Pseudomonas sp. |
| GM45 | 2 | GM | Burkholderia sp. |
| GM46 | 2 | GM | Stenotrophomonas sp. |
| GM47 | 2 | GM | Pseudomonas sp. |
| GM48 | 2 | GM | Pseudomonas sp. |

| | | | |
|---|---|---|---|
| GM49 | 2 | GM | Pseudomonas sp. |
| GM50 | 2 | GM | Luteibacter sp. |
| GM51 | 2 | GM | Klebsiella sp. |
| GM53 | 2 | GM | Burkholderia sp. |
| GM54 | 2 | GM | Burkholderia sp. |
| GM55 | 2 | GM | Burkholderia sp. |
| GM57 | 2 | GM | Burkholderia sp. |
| GM58 | 2 | GM | Rhizobium sp. |
| GM59 | 2 | GM | Pantoea sp. |
| GM60 | 2 | GM | Luteibacter sp. |
| GM40.1 | 2 | GM | unidentified |
| GM45.1 | 2 | GM | unidentified |
| BV1 | 1 | BV | Stenotrophomonas sp. |
| BV2 | 1 | BV | Achromobacter sp. |
| BV3 | 1 | BV | Pseudomonas sp. |
| BV4 | 1 | BV | Stenotrophomonas sp. |
| BV5 | 1 | BV | Stenotrophomonas sp. |
| BV6 | 1 | BV | Pseudomonas sp. |
| BV7 | 1 | BV | Pseudomonas sp. |
| BV8 | 1 | BV | Xanthomonas sp. |
| BV9 | 1 | BV | Pseudomonas sp. |
| BV10 | 1 | BV | Salmonella sp. |
| BV11 | 1 | BV | Herbaspirillum sp. |
| BV12 | 1 | BV | Pseudomonas sp. |
| BV13 | 1 | BV | Herbaspirillum sp. |
| BV14 | 1 | BV | Herbaspirillum sp. |
| BV15 | 1 | BV | Xanthomonas sp. |
| BV16 | 1 | BV | Enterobacter sp. |
| BV17 | 1 | BV | Enterobacter sp. |
| BV18 | 1 | BV | Xanthomonas sp. |
| BV20 | 1 | BV | Herbaspirillum sp. |
| BV21 | 1 | BV | Pantoea sp. |

| | | | |
|---|---|---|---|
| BV22 | 1 | BV | Enterobacter sp. |
| BV23 | 1 | BV | Shigella sp. |
| BV24 | 1 | BV | Shigella sp. |
| BV25 | 1 | BV | Shigella sp. |
| BV26 | 1 | BV | Pantoea sp. |
| BV27 | 1 | BV | Pantoea sp. |
| BV28 | 1 | BV | Pantoea sp. |
| BV29 | 1 | BV | Shigella sp. |
| BV30 | 1 | BV | Shigella sp. |
| BV23.1 | 1 | BV | Enterobacter sp. |
| BV31 | 2 | BV | Chryseobacterium sp. |
| BV32 | 2 | BV | Pseudomonas sp. |
| BV33 | 2 | BV | Pantoea sp. |
| BV34 | 2 | BV | Salmonella sp. |
| BV35 | 2 | BV | Enterobacter sp. |
| BV36 | 2 | BV | Kluyvera sp. |
| BV37 | 2 | BV | Salmonella sp. |
| BV38 | 2 | BV | Salmonella sp. |
| BV40 | 2 | BV | Enterobacter sp. |
| BV43 | 2 | BV | Pantoea sp. |
| BV45 | 2 | BV | Burkholderia sp. |
| BV47 | 2 | BV | Burkholderia sp. |
| BV49 | 2 | BV | Burkholderia sp. |
| BV50 | 2 | BV | Dyella sp. |
| BV52 | 2 | BV | Burkholderia sp. |
| BV53 | 2 | BV | Burkholderia sp. |
| BV56 | 2 | BV | Burkholderia sp. |
| BV57 | 2 | BV | Burkholderia sp. |
| BV59 | 2 | BV | Enterobacter sp. |
| BV60 | 2 | BV | Burkholderia sp. |
| BV40.1 | 2 | BV | Enterobacter sp. |

[1]Name: isolate identification.

[2]Niche: 1 = roots; 2 = rhizospheric soil.

[3]Sampling site: JC = Júlio de Castilhos, SB = São Borja, VA = Vacaria, CM = Campina das Missões, GM = Guarani das Missões, and BV = Boa Vista do Cadeado.

[4]Genera: according to partial 16S rRNA gene sequencing.
